# Multiples complications dans les suites opératoires d’une prothèse totale de hanche: à propos d’un cas

**DOI:** 10.11604/pamj.2025.51.68.48073

**Published:** 2025-07-08

**Authors:** Johnny Cizemba, Kevin Ndangi, Pistis Zamba, Raymond Minkutu, Chris Muteba, Doudou Diumu

**Affiliations:** 1 Service d'Orthopédie et Traumatologie, Centre Hospitalier de Basse Terre, Guadeloupe, France,; 2 Département de Chirurgie, Cliniques Universitaires de Kinshasa, Kinshasa, République Démocratique du Congo

**Keywords:** Prothèse, hanche, multiples complications, acceptable évolution, cas clinique, Prosthesis, hip, multiple complications, acceptable evolution, case report

## Abstract

Nous présentons le cas d'un patient de 70 ans, opéré pour une fracture du col du fémur gauche Garden 4. Le traitement avait consisté en une prothèse totale de hanche (PTH) primaire dont l'évolution à court terme était marquée par 6 complications, ayant nécessité 3 reprises chirurgicales. La disponibilité des moyens logistiques, la rapidité dans les gestes ont permis d'obtenir une issue acceptable.

## Abstract (en)

We present the case of a 70-year-old patient, operated for a fracture of the neck of the left femur classified as Garden 4. The treatment consisted of a primary total hip prosthesis whose short-term evolution was marked by 6 complications requiring 3 surgical revisions. The availability of logistical means and the speed of the gestures made it possible to obtain an acceptable outcome.

## Introduction

La PTH est une technique en perpétuel accroissement. Elle apporte une autonomie rapide et une indolence dans le traitement des affections aiguës et chroniques de la hanche [1]. Cependant, cette chirurgie prothétique expose au risque de la survenue des complications qui peuvent engager le pronostic fonctionnel et /ou vital [2,3].

Nous rapportons un cas de multiples complications, multiples reprises survenues dans les suites opératoires d'une PTH primaire gauche mais d'évolution heureuse. Notre objectif est de présenter notre approche ainsi que les résultats obtenus.

## Patient et observation

**Information du patient:** monsieur AD, âgé de 70 ans, admis aux urgences du Centre Hospitalier de Basse Terre (CHBT) pour une douleur et une impotence fonctionnelle du membre inférieur gauche. Ses antécédents étaient non contributifs à la pathologie actuelle. L'échelle visuelle analogique (EVA) était à 8/10 et son score de Parker était estimé à 9. Aucun traitement n'avait été entrepris avant son admission aux urgences.

**Résultats cliniques:** à l'examen physique, le membre inférieur gauche présentait un raccourcissement, une déviation latérale du pied et une douleur à la mobilisation de la hanche.

**Chronologie de l'épisode actuel:** le patient a bénéficié d'une arthroplastie de la hanche gauche sans incident. La sortie de l'hôpital est autorisée au deuxième jour. Il a été réadmis deux jours après dans un contexte de douleur après une chute. Les radiographies révèlent une luxation associée à une fracture periprothétique. Une deuxième opération est réalisée. Une deuxième luxation a eu lieu 3 jours après la reprise chirurgicale. Une réduction à foyer fermé est obtenue. Un écoulement anormal est observé 10 jours après la reprise. Un lavage articulaire est réalisé.

**Figure 1:**
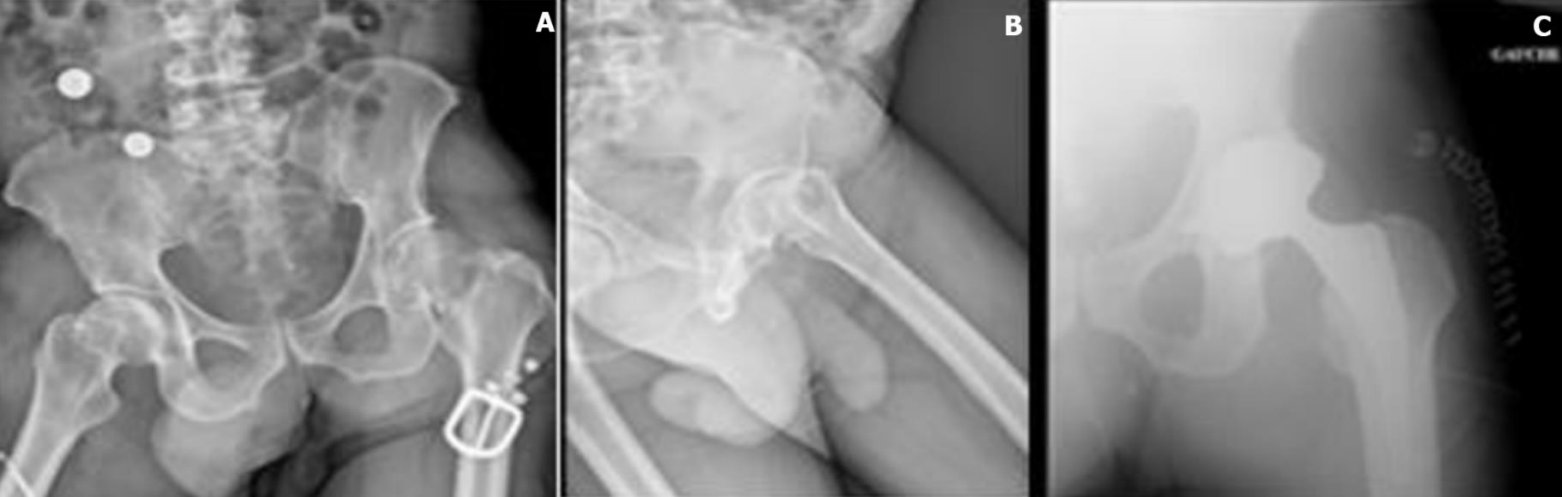
(A,B) radiographies préopératoires et (C) postopératoire

**Démarche diagnostique:** les radiographies de la hanche gauche et du bassin montraient une fracture du col fémoral gauche classée Garden 4 (Figure 1). Le bilan biologique était dans les normes.

**Intervention thérapeutique:** nous avons réalisé une arthroplastie totale de hanche à double mobilité type capitol-T du laboratoire medacta (tige taille 2 sans ciment, col court, tête 28 et cupule 50) par voie postérieure. Le patient était sorti au troisième jour postopératoire vers un centre de rééducation.

**Figure 2:**
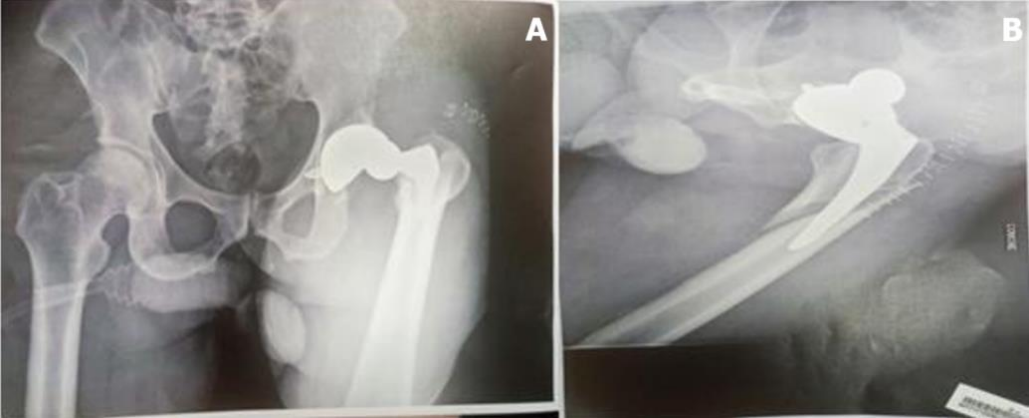
(A) luxation et (B) fracture periprothétique

**Figure 3:**
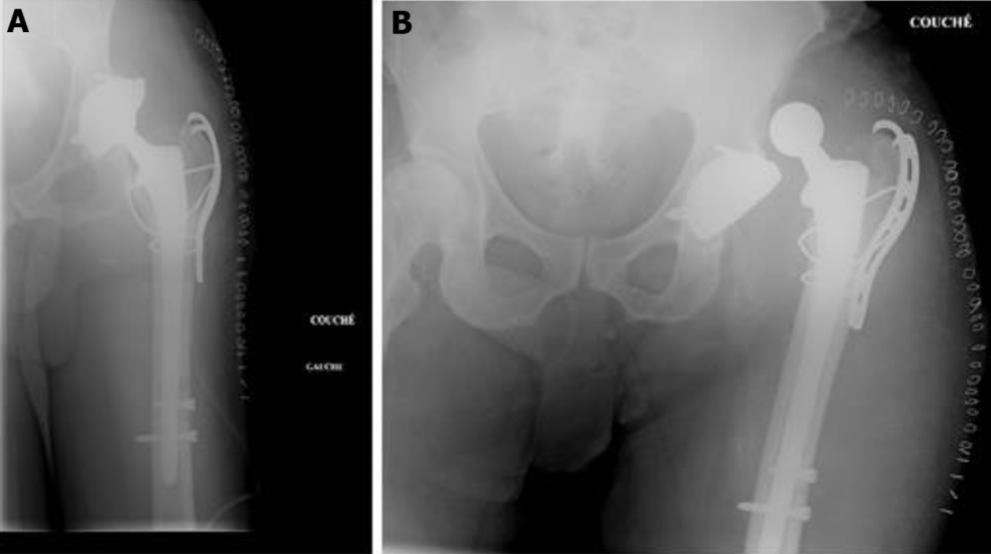
reprise prothétique, tige longue et plaque Dall-Miles (A) et deuxième luxation (B)

**Suivi et résultats:** l'évolution était marquée par une chute au deuxième jour de son admission au centre. Le patient a été réadmis dans notre service dans un tableau des douleurs. La radiographie du bassin réalisée, montrait une luxation postérieure de la prothèse associée à une fracture periprothétique type B2 de Vancouver (Figure 2). Nous n'avons pas eu recours au scanner pour confirmer le diagnostic. Nous avons réalisé un changement de la tige fémorale, une synthèse de la fracture à l'aide d'une plaque courte de Dall-Miles (Figure 3). L'évolution était marquée au troisième jour de la deuxième intervention par la survenue d'une nouvelle luxation sans descellement sur les versants fémoral et acétabulaire. Une réduction était obtenue sans difficulté sous anesthésie générale.

**Figure 4:**
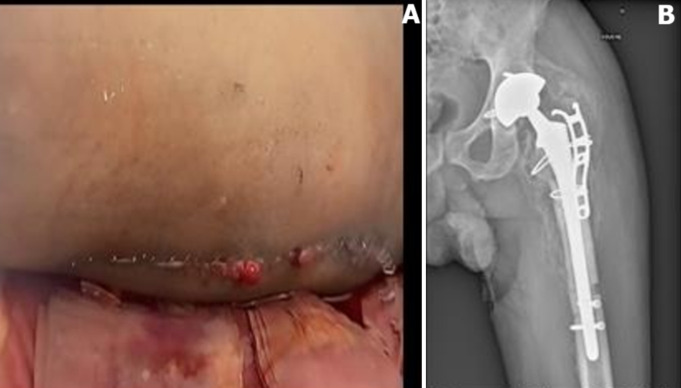
infection du site opératoire (A) prothèse en place (B)

Dix jours après la première intervention, nous avons noté l'issue des sécrétions sero-purulantes (Figure 4). Le diagnostic d'une infection précoce était retenu. Nous avons procédé au prélèvement bactériologique, à un lavage articulaire et au changement des pièces mobiles. Les prélèvements bactériologiques avaient permis de mettre en évidence deux germes: *Enterococcus faecalis et Pseudomonas aeruginosa*.

Une antibiothérapie faite de l'Augmentin et de la ciprofloxacine a été instaurée pour une durée de 6 semaines. À deux semaines de l'antibiothérapie, le patient a présenté une autre thrombose veineuse superficielle de l'avant-bras droit. Cette dernière complication n'avait pas de lien direct avec la prothèse. L'évolution était favorable (arrêt d'écoulement, normalisation de la biologie et reprise de la rééducation) et la durée d'hospitalisation était longue (30 jours). La radiographie à la sortie permettait de voir une ossification des parties molles avec un début de formation de la cal.

**Point de vue du patient:** il était satisfait de la prise en charge malgré les multiples complications.

**Consentement éclairé:** les auteurs ont obtenu le consentement éclairé du patient pour la publication de cet article.

## Discussion

La mise en place d'une PTH est actuellement le *gold standard* du traitement de l'arthrose de hanche sévère en cas d'échec de traitement médical. Malgré les avantages qu'elle offre, le patient reste exposé pendant toute sa vie au risque de réintervention suite à une complication sur sa prothèse [4]. Ce chapitre discute des suites opératoires d'une PTH marquée par 6 épisodes de complications. Il s'agit de deux luxations, d'une fracture periprothétique, d'une infection, d'une calcification periprothétique et d'une thrombose veineuse. La luxation sur prothèse reste la plus décrite. Elle peut être isolée ou associée à des fractures. Elle peut être due aux facteurs liés au patient ou à la technique. Il en est de même pour les fractures periprothétiques [5,6].

Dans notre cas, plusieurs facteurs ont été mis en évidence. La chute, le positionnement du métal back qui montre un excès de couverture, responsable d'un effet came avec conflit (impingement), l'absence de la restauration de l'offset fémoral (Figure 1C) et le varus de la tige (Figure 1C). Deux formes de traitement sont évoquées en présence d'une fracture periprothétique: le traitement conservateur et le traitement chirurgical (ostéosynthèse seule et le remplacement prothétique avec ou sans ostéosynthèse) [7]. Pour ce qui nous concerne, nous avons réalisé le remplacement de la prothèse avec ostéosynthèse. L'infection s'est déclarée 10 jours après la première intervention (Figure 4A). Une infection précoce (< 30 jours) révèle habituellement une infection à pathogène virulent comme les *Staphylococcus aureus*, Streptocoques ß-hémolytiques ou plus rarement par Entérobactéries [8].

*Enterococcus faecalis* et le *Pseudomonas aeruginosa* multi sensibles ont été retrouvés. La normalisation de la protéine C-réactive (CRP) était obtenue au bout de cinq semaines d'antibiothérapie. La survenue de la thrombose au membre supérieur était liée au midline et avait nécessité l'instauration d'une héparinothérapie à dose curative. Il faut noter toutefois que, les chirurgies orthopédiques sont à très haut risque d'évènements thrombo-emboliques. Leur localisation préférentielle étant le membre inférieur [9].

La dernière complication notée était une ossification des parties molles souvent liée au traumatisme chirurgical infligé aux parties molles (Figure 4B). L'évolution était favorable pour l'ensemble des complications. Le patient était sorti de l'hôpital vers un centre de rééducation après un séjour de 30 jours.

## Conclusion

Les complications liées aux PTH sont souvent décrites de manière isolée et/ou sur de grands échantillons de patients. Ce cas a l'avantage de présenter en même temps plusieurs complications sur un seul patient avec une évolution favorable.
